# Prevalence of Low Back Pain and Its Associated Factors Among Medical Students at Nineveh University in Iraq: A Cross-Sectional Study

**DOI:** 10.7759/cureus.65770

**Published:** 2024-07-30

**Authors:** Abdulhameed Alhadeethi, Hosny Elkhawaga, Mohamed H Khalil, Ahmed A Basheer

**Affiliations:** 1 College of Medicine, Ninevah University, Mosul, IRQ; 2 Department of General Medicine, Al-Salam Teaching Hospital, Mosul, IRQ; 3 Medical Research Group of Egypt, Negida Academy, Arlington, USA; 4 Faculty of Physical Therapy, Cairo University, Giza, EGY; 5 Faculty of Medicine, Zagazig University, Zagazig, EGY; 6 Department of Physical Therapy for Musculoskeletal Disorders, Faculty of Physical Therapy, Beni Suef University, Beni Suef, EGY

**Keywords:** low back pain (lbp), orthopedics and physical therapy, cross-sectional study, lumbar spine pain, spine injuries, musculoskeletal diseases, prevalence, orthopedic disease, iraq, occupational health hazard

## Abstract

Background

Low back pain (LBP) is a common problem encountered in medical practice, leading to limitations in daily activities and causing social and economic hardships.

Objectives

This study aimed to assess the prevalence of LBP and its associated factors among medical students at Nineveh University in Iraq.

Methods

Between December 2022 and January 2023, a cross-sectional study was conducted among medical students at Nineveh University. A modified version of the Standard Nordic Questionnaire was used for data collection.

Results

Out of 308 students, 229 (74.4%) experienced LBP at some point in their lives. In addition, 209 (67.9%) reported having LBP during the last 12 months, 148 (48.1%) during the previous seven days, and 126 (40.9%) at the time of answering the survey. Factors significantly associated with LBP during the last 12 months were being in the fifth-stage academic year (p=0.047), family history of LBP (p=0.003), and history of trauma (p=0.006). On the multivariable logistic regression analysis, factors significantly associated with LBP during the last 12 months were family history of LBP (p=0.02) and history of trauma (p=0.01).

Conclusions

The prevalence of LBP among medical students at Nineveh University was comparatively high. A family history of LBP and a history of trauma were factors significantly associated with LBP during the last 12 months. Managing this health concern should be a priority for the administration of medical schools.

## Introduction

Low back pain (LBP) is a common health problem that has been reported to affect 50-80% of individuals at least once in their lifetime and about 18% at any given time [1-3]. LBP is a cause of activity limitation and adversely affects quality of life [4]. Additionally, LBP has growing economic burdens due to loss of productivity and the cost of treatment [5].

Several risk factors have been reported to influence the occurrence of LBP, including increased physical stress on the spine, psychological stress, and poor general health [6]. Furthermore, stress, body position at work, and lack of physical activity have been identified as risk factors among healthcare providers [7]. Regarding medical students, in the final year of study, anxiety, high mental pressure, or psychological distress have been reported to be associated with LBP [8].

Medical students encounter highly demanding curricula throughout their academic years, which may expose them to stress and a sedentary lifestyle due to long hours of lectures, practical training, and studying. The stress and sedentary lifestyle put them at risk of developing LBP [6,9]. Because LBP can affect their academic performance and future jobs, it is an important problem that needs to be studied among medical students. It is worth noting that several studies have investigated LBP among medical students in different countries [8]. However, to the best of our knowledge, no studies have been performed on this health condition in Iraq before. Therefore, this study aimed to assess the prevalence of LBP and its associated factors among medical students at Nineveh University in Iraq.

## Materials and methods

Study design and ethics

This study adopted an observational approach employing a cross-sectional design with a convenient sample, adhering to the guidelines and prerequisites outlined in the Strengthening the Reporting of Observational Studies in Epidemiology (STROBE) statement [10]. This study was conducted in strict accordance with the principles outlined in the Helsinki Declaration of the World Medical Association [11]. The Deanship of the College of Medicine, Nineveh University, Iraq, approved this study. Online, informed consent was obtained from all participants.

Setting and participants

This study was carried out at Nineveh University in Mosul City, situated in the northern region of Iraq, between December 2022 and January 2023. The participants of the study were male and female medical students from the first to the sixth stage, while medical interns were excluded.

Instruments

We used an online, self-administered survey conducted via Google Forms (Google LLC, Mountain View, CA, USA) to gather data. The questionnaire was designed in English and was an adapted variant of the standardized Nordic questionnaire [12]. This adaptation was performed from previous studies [13,14] and specifically tailored for use among medical students, with a particular focus on assessing LBP. Before being used in the study, the questionnaire was piloted with five students to ensure its suitability.

The questionnaire had three sections: (i) summarize the survey's purpose, aim, and target participants; (ii) include demographic data (sex, age, academic years, height, and weight), lifestyle-related questions (physical exercise, studying hours in a day, smoking or not, type of participant activity), family history of LBP, and history of trauma; and (iii) investigate the prevalence of LBP during the last 12 months and its related questions, which include duration, if LBP reduces his activity, and if LBP makes the participant see a doctor. Additionally, questions about the prevalence of LBP during the time of answering the survey (point prevalence), during the previous seven days, and at some point in life (lifetime prevalence) were included in this section.

Sample size calculation

Study size calculation was handled using OpenEpi version 3.01 (www.openepi.com). Under a 95% confidence level, 0.05 margin of error, 1,651 total population, and 33% prevalence of LBP based on a study conducted among medical students at Taif University in Saudi Arabia [15], a sample of 282 participants was considered a minimum sample.

Statistical analysis

Data analyses were conducted using jamovi version 2.4.11, which is accessible for use at the website (www.jamovi.org). In our descriptive analysis, the continuous variables were summarized using the median and IQR, while the categorical variables were presented as proportions and percentages. The chi-squared test and univariate logistic regression were first used to determine the association and ORs and 95% CIs for factors associated with LBP. Mann-Whitney was conducted to identify the difference for the non-normally distributed variables (studying hours in a day). Significant variables with a p-value less than 0.05 were included in the multivariate logistic regression analysis. All statistical analyses were performed with a significance level set at p<0.05 and a confidence interval of 95%.

## Results

Study participants

**Figure 1:**
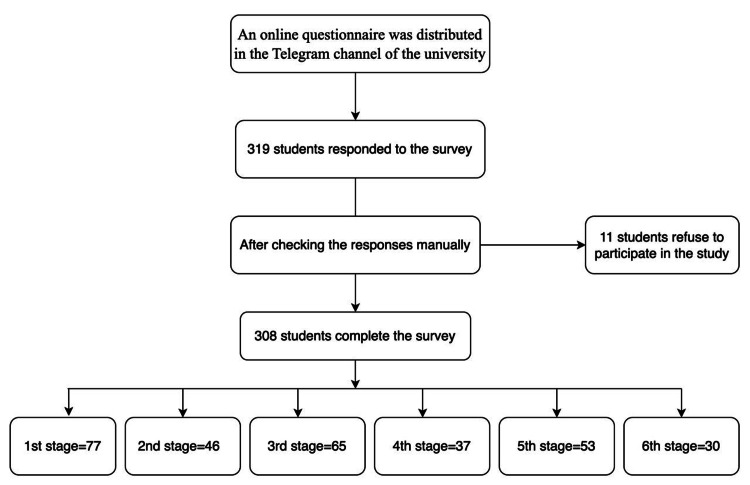
Questionnaire distribution flow chart of the sample population

Of the 319 students who responded to the survey, 308 (96.6%) agreed to participate in the study, and 11 (3.4%) refused. Figure 1 shows the questionnaire distribution flow chart. There were 180 (58.4%) females and 128 (41.6%) males. The ages of the participants were ≤21 years in 175 (56.8%), 22-24 years in 84 (27.3%), and ≥25 years in 49 (15.9%). Moreover, 204 (66.2%) participants had a BMI ≤25, the majority (267, 86.7%) never smoked, and 45 (14.6%) performed regular exercise. A summary of sociodemographic and lifestyle factors is shown in Table 1.

**Table 1:** Sociodemographic and lifestyle factors of the study participants BMI: body mass index, LBP: low back pain, IQR: interquartile range

	Total (N=308)	Female (N=180)	Male (N=128)
Age group (years)			
≤21	175 (56.8%)	108 (60%)	67 (52.3%)
22-24	84 (27.3%)	55 (30.6%)	29 (22.7%)
≥25	49 (15.9%)	17 (9.4%)	32 (25%)
Academic year			
1st stage	77 (25%)	39 (21.7%)	38 (29.7%)
2nd stage	46 (14.9%)	30 (16.7%)	16 (12.5%)
3rd stage	65 (21.1%)	45 (25.0%)	20 (15.6%)
4th stage	37 (12%)	21 (11.7%)	16 (12.5%)
5th stage	53 (17.2%)	33 (18.3%)	20 (15.6%)
6th stage	30 (9.7%)	12 (6.7%)	18 (14.1%)
BMI (kg/m²)			
≤25	204 (66.2%)	127 (70.6%)	77 (60.2%)
>25	104 (33.8%)	53 (29.4%)	51 (39.8%)
Smoking status			
Never smoked	267 (86.7%)	177 (98.3%)	90 (70.3%)
Ex-smoker	13 (4.2%)	1 (0.6%)	12 (9.4%)
Smoker	28 (9.1%)	2 (1.1%)	26 (20.3%)
Exercise			
Not at all	130 (42.2%)	80 (44.4%)	50 (39.1%)
Not regular	133 (43.2%)	78 (43.3%)	55 (43%)
Regular	45 (14.6%)	22 (12.2%)	23 (18%)
Most activity is done by			
No specific position for a long time	62 (20.1%)	35 (19.4%)	27 (21.1%)
Bending	26 (8.4%)	16 (8.9%)	10 (7.8%)
Sitting	134 (43.5%)	80 (44.4%)	54 (42.2%)
Walking or standing	86 (27.9%)	49 (27.2%)	37 (28.9%)
Family history of LBP			
No	143 (46.4%)	76 (42.2%)	67 (52.3%)
Yes	165 (53.6%)	104 (57.8%)	61 (47.7%)
History of trauma			
No	271 (88.0%)	159 (88.3%)	112 (87.5%)
Yes	37 (12.0%)	21 (11.7%)	16 (12.5%)
Studying hours in a day			
Median (IQR)	4 (3)	5 (3)	4 (2)

Prevalence of LBP

**Figure 2:**
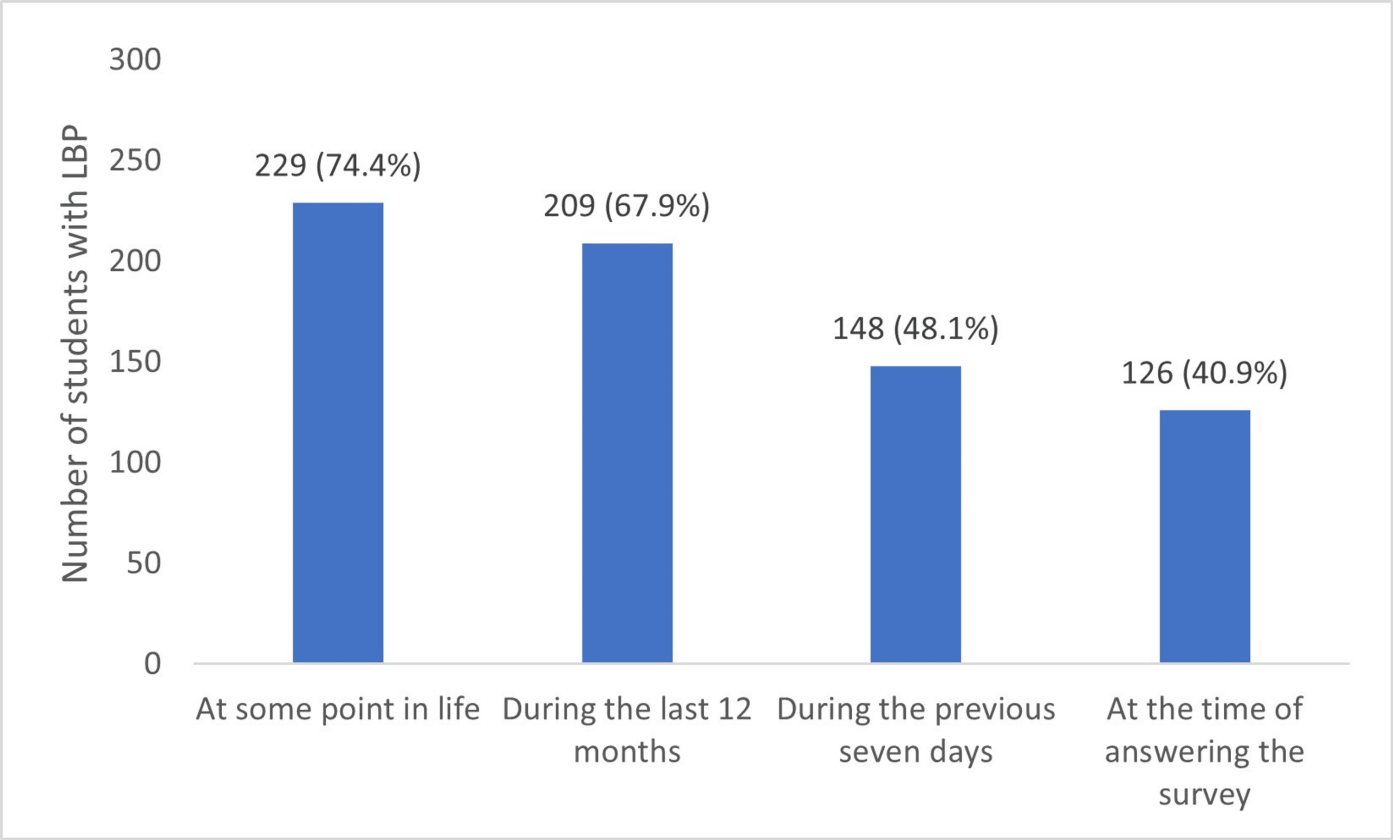
Prevalence of LBP at different times LBP: low back pain

Out of 308 students, 229 (74.4%) experienced LBP at some point in their lives. In addition, 209 (67.9%) reported having LBP during the last 12 months, 148 (48.1%) during the previous seven days, and 126 (40.9%) at the time of answering the survey, as shown in Figure 2.

Of a total of 209 students who reported LBP during the last 12 months, 119 (56.94%) mentioned that their pain duration was between one and seven days, 28 (13.4%) between eight and 30 days, and 27 (12.92%) more than 30 days, and 19 (9.09%) reported that their pain was every day, and 16 (7.66%) reported that their pain was less than one day. Moreover, 111 (53.11%) reported a reduction in their activity, and 33 (15.79%) had seen a doctor.

Factors associated with LBP during the last 12 months

Factors associated with LBP during the last 12 months were examined separately using univariate and multivariate logistic regression, as shown in Table 2. Being in the fifth stage of the academic year (OR=2.19, 95% CI (1.01-4.74), p=0.047), family history of LBP (OR=2.06, 95% CI (1.27-3.35), p=0.003), and history of trauma (OR=4.45, 95% CI (1.53-12.95), p=0.006) were found to be significantly associated with LBP during the last 12 months. Students with LBP during the last 12 months showed significantly shorter daily studying hours (median=4, IQR=3) compared to those without LBP (median=5, IQR=2) (p=0.03).

**Table 2:** Factors associated with LBP during the last 12 months using univariate and multivariate logistic regression (N=308) ^*^ indicates significant differences (p<0.05) BMI: body mass index, LBP: low back pain, IQR: interquartile range, OR: odds ratio, CI: confidence interval

Variables	Yes (N=209)	No (N=99)	Univariate analysis		Multivariable analysis	
			OR (95% CI)	p	OR (95% CI)	p
Gender						
Male	82 (64.1%)	46 (35.9 %)	Ref			
Female	127 (70.6%)	53 (29.4%)	1.34 (0.83-2.18)	0.23	-	-
Age group (years)						
≤21	116 (66.3%)	59 (33.7%)	Ref			
22-24	61 (72.6%)	23 (27.4%)	1.35 (0.76-2.39)	0.31	-	-
≥25	32 (65.3%)	17 (34.7%)	0.96 (0.49-1.86)	0.9	-	-
Academic year						
1st stage	45 (58.4%)	32 (41.6 %)	Ref			
2nd stage	34 (73.9%)	12 (26.1%)	2.01 (0.91-4.48)	0.09	1.82 (0.79-4.19)	0.16
3rd stage	46 (70.8%)	19 (29.2 %)	1.72 (0.85-3.47)	0.13	1.64 (0.79-3.41)	0.19
4th stage	22 (59.5%)	15 (40.5%)	1.04 (0.47-2.32)	0.92	1.03 (0.45-2.36)	0.95
5th stage	40 (75.5%)	13 (24.5 %)	2.19 (1.01-4.74)	0.047^*^	2.2 (0.99-4.89)	0.054
6th stage	22 (73.3%)	8 (26.7%)	1.96 (0.77-4.94)	0.16	1.64 (0.62-4.3)	0.32
BMI (kg/m²)						
≤25	134 (65.7%)	70 (34.3 %)	Ref			
>25	75 (72.1%)	29 (27.9%)	1.35(0.81-2.27)	0.25	-	-
Smoking status						
Never smoked	182 (68.2%)	85 (31.8 %)	Ref			
Ex-smoker	10 (76.9%)	3 (23.1%)	1.56 (0.42-5.8)	0.51	-	-
Smoker	17 (60.7%)	11 (39.3%)	0.72 (0.32-1.61)	0.42	-	-
Exercise						
Not at all	88 (67.7%)	42 (32.3 %)	Ref			
Not regular	94 (70.7%)	39 (29.3%)	1.15 (0.68-1.94)	0.6	-	-
Regular	27 (60%)	18 (40 %)	0.72 (0.36-1.44)	0.35	-	-
Most activity is done by						
No specific position for a long time	41 (66.1%)	21 (33.9%)	Ref			
Bending	41.0 (69.2%)	8 (30.8%)	1.15 (0.43-3.09)	0.78	-	-
Sitting	99 (73.9%)	35 (26.1%)	1.45 (0.76-2.78)	0.27	-	-
Walking or standing	51 (59.3%)	35 (40.7 %)	0.75 (0.38-1.47)	0.4	-	-
Family history of LBP						
No	85.0 (59.4%)	58 (40.6 %)	Ref			
Yes	124.0 (75.2%)	41 (24.8%)	2.06 (1.27-3.35)	0.003^*^	1.86 (1.12-3.1)	0.02^*^
History of trauma						
No	176.0 (64.9%)	95 (35.1 %)	Ref			
Yes	33.0 (89.2%)	4 (10.8%)	4.45 (1.53-12.95)	0.006^*^	4.05 (1.37-11.99)	0.01^*^
Studying hours in a day, median (IQR)	4 (3)	5 (2)		0.03^*^	0.91 (0.82-1)	0.057

On the multivariable logistic regression analysis, factors significantly associated with LBP during the last 12 months were family history of LBP (OR=1.86, 95% CI (1.12-3.1), p=0.02) and history of trauma (OR=4.05, 95% CI (1.37-11.99), p=0.01).

Factors associated with LBP during the previous seven days

Factors associated with LBP during the previous seven days were examined separately using univariate and multivariate logistic regression, as shown in Table 3. BMI greater than 25 kg/m² (OR=1.91, 95% CI (1.18-3.08), p=0.008), performing regular exercise (OR=0.33, 95% CI (0.16-0.7), p=0.004), and family history of LBP (OR=1.67, 95% CI (1.06-2.62), p=0.03) were found to be significantly associated with LBP during the previous seven days. Students with LBP during the previous seven days showed significantly longer daily studying hours (median=5, IQR=2) compared to those without LBP (median=4, IQR=3) (p=0.04).

**Table 3:** Factors associated with LBP during the previous seven days using univariate and multivariate logistic regression (N=308) ^*^ indicates significant differences (p<0.05) BMI: body mass index, LBP: low back pain, IQR: interquartile range, OR: odds ratio, CI: confidence interval

Variables	Yes (N=148)	No (N=161)	Univariate analysis		Multivariable analysis	
			OR (95% CI)	p	OR (95% CI)	p
Gender						
Male	55 (43%)	73 (57 %)	Ref			
Female	93 (51.7%)	87(48.3 %)	1.42 (0.89-2.24)	0.13	-	-
Age group (years)						
≤21	90 (51.4%)	85 (48.6%)	Ref			
22-24	37 (44%)	47 (56%)	0.74 (0.44-1.25)	0.27	-	-
≥25	21 (42.9%)	28 (57.1%)	0.71 (0.37-1.34)	0.29	-	-
Academic year						
1st stage	31 (40.3%)	46 (59.7%)	Ref			
2nd stage	24 (52.2%)	22 (47.8%)	1.62 (0.78-3.38)	0.2	-	-
3rd stage	36 (55.4%)	29 (44.6 %)	1.84 (0.94-3.59)	0.07	-	-
4th stage	19 (51.4%)	18 (48.6%)	1.57 (0.71-3.45)	0.26	-	-
5th stage	26 (49.1%)	27 (50.9 %)	1.43 (0.71-2.89)	0.32	-	-
6th stage	12 (40%)	18 (60 %)	0.99 (0.42-2.34)	0.98	-	-
BMI (kg/m²)						
≤25	87 (42.6%)	117 (57.4%)	Ref			
>25	61(58.7%)	43 (41.3%)	1.91 (1.18-3.08)	0.008^*^	1.81 (1.11-2.96)	0.02^*^
Smoking status						
Never smoked	127(47.6%)	140 (52.4 %)	Ref			
Ex-smoker	8.0 (61.5%)	5 (38.5%)	1.76 (0.56-5.53)	0.33	-	-
Smoker	13.0 (46.4%)	15 (53.6%)	0.96 (0.44-2.09)	0.91	-	-
Exercise						
Not at all	68 (52.3%)	62 (47.7%)	Ref			
Not regular	68 (51.1%)	65 (48.9 %)	0.95 (0.59-1.55)	0.85	0.99 (0.61-1.63)	0.98
Regular	12 (26.7%)	33 (73.3 %)	0.33 (0.16-0.7)	0.004^*^	0.37 (0.17-0.794)	0.01^*^
Most activity is done by						
No specific position for a long time	28 (45.2%)	34 (54.8 %)	Ref			
Bending	10 (38.5%)	16 (61.5 %)	0.76 (0.3-1.93)	0.56	-	-
Sitting	79 (59%)	55 (41 %)	1.74 (0.95-3.2)	0.07	-	-
Walking or standing	31 (36%)	55 (64 %)	0.68 (0.35-1.33)	0.27	-	-
Family history of LBP						
No	59 (41.3%)	84 (58.7 %)	Ref			
Yes	89 (53.9%)	76 (46.1%)	1.67 (1.06-2.62)	0.03^*^	1.53 (0.96-2.43)	0.07
History of trauma						
No	128 (47.2%)	143 (52.8%)	Ref			
Yes	20 (54.1%)	17 (45.9 %)	1.31 (0.66-2.62)	0.43		
Studying hours in a day, median (IQR)	5 (2)	4 (3)		0.04^*^	1.07 (0.98-1.18)	0.15

On the multivariable logistic regression analysis, factors significantly associated with LBP during the previous seven days were BMI greater than 25 kg/m² (OR=1.81, 95% CI (1.11-2.96), p=0.02) and performing regular exercise (OR=0.37, 95% CI (0.17-0.794), p=0.01).

## Discussion

Our study marked the first effort to assess LBP among medical students in Iraq. This study aimed to assess LBP prevalence and its associated factors in a sample of medical students at Nineveh University. Our study showed that the prevalence of LBP at the time of answering the survey, during the previous seven days, during the last 12 months, and at some point in life were 40.9%, 48.1%, 67.9%, and 74.4%, respectively. The prevalence of LBP during the last 12 months in our study (67.9%) was similar to the prevalence reported in previous studies conducted in Saudi Arabia (67%) [16] and (61.4%) [17], Bangladesh (63.3%) [14], Jordan (63.1%) [18], Brazil (59.9%) [19], and Serbia (59.5%) [20]. On the other hand, the lower prevalence was reported in studies conducted in India (47.5%) [21], Malaysia (46.1%) [13], the USA (42.8%) [22], China (40.1%) [23], and Pakistan (38.6%) [24].

Stoicism and resilience in the face of adversity are often valued traits in Iraqi culture. This cultural norm may lead individuals, including medical students, to underreport symptoms of pain or to endure discomfort without seeking medical help. The perception that enduring pain without complaint is a sign of strength can result in a high threshold for reporting LBP. Additionally, there may be a stigma associated with admitting to health issues, especially in a competitive and demanding field such as medicine, where students might fear being perceived as weak or less capable. However, Iraqi medical students reported a relatively high prevalence of LBP, which reflects the extent of the impact of the problem in the presence of this cultural norm.

The findings of our study revealed that gender was not associated with the prevalence of LBP during the last 12 months and during the previous seven days, which is in agreement with previous studies [23,24]. Conversely, other studies indicated that the prevalence of LBP increases with female gender [25,26]. Notably, our study found that fifth-year students had a greater prevalence of LBP during the last 12 months than other students. The explanation of this finding may be that fifth-year students at any Iraqi medical school are exposed to significantly longer study hours, hospital training, and demanding routines than students at other academic levels.

Although overweight and obesity have been reported to be risk factors for LBP [27], our study demonstrated no association between BMI and the prevalence of LBP during the last 12 months. On the other hand, our study showed that a BMI of more than 25 kg/m² showed an association with LBP during the previous seven days. Furthermore, although evidence has suggested that smoking is associated with LBP [6], we found no association between smoking status and LBP during the last 12 months and during the previous seven days. Our study showed no association between participant activity patterns and LBP during the last 12 months and during the previous seven days. However, students performing regular exercise had a lower prevalence of LBP during the previous seven days than other students. The explanation for this finding may be that physical activity is associated with a lower prevalence of LBP because it has several benefits related to physiological health [28,29].

Interestingly, our study showed that participants whose family members have suffered from LBP in the past and those who have suffered from trauma have a higher chance of suffering from LPB in the future. However, the details about the family history, as well as the nature of the trauma and its complications, are not known. Notably, students with LBP during the last 12 months showed shorter daily studying hours compared to those without LBP, which may reflect the negative effect of LBP on the quality of academic life, academic performance, and achievement. Conversely, students with LBP during the previous seven days showed longer daily studying hours compared to those without LBP, which reflects the association between long studying hours and LBP.

Strengths and limitations

To our knowledge, this is the first study undertaken at Ninevah University and in the country on this topic; as such, it contributes to the scientific community with valuable new information. However, cross-sectional studies are limited in demonstrating causation or defining temporal correlations since they only record a single snapshot of data at a particular point in time. Additionally, convenient sampling may not accurately represent the population of interest and may lead to selection bias. Participants who are more willing to participate in the study may have different characteristics from those who are not, which can limit the generalizability of the results.

Recommendations

Overall, LBP is an important problem among medical students that needs more attention. Managing this health concern should be a priority for the administration of medical schools. Students’ education, advice to be more physically active, and performing regular exercise may be preventive measures against LBP [30]. The administration of medical schools should provide workshops about LBP prevention and management and periodically host sports activities.

## Conclusions

The prevalence of LBP among medical students at Nineveh University was comparatively high. Family history of LBP and a history of trauma were factors significantly associated with LBP during the last 12 months. Managing this health concern should be a priority for the administration of medical schools. Students' education about LBP prevention and management is recommended.
